# The Summer Conventions

**Published:** 1902-06

**Authors:** 


### Editorial.

### THE SUMMER CONVENTIONS.

THE annual gatherings that will convene in the coming two months in this country and in Europe are not only very important, but they indicate, in some respects, a decided advance over anything that has preceded them in the line of organized effort. The indications are that all of them will be occupied with important matters that intimately concern the welfare and progress of dentistry.

The Section on Stomatology of the American Medical Association, which meets June 10 to 13, at Saratoga Springs, promises to be an interesting meeting, if we may judge by the programme published. This, the first of the National organizations, is to be followed, July 24, 25, and 28, by, respectively, the National Association of Dental Faculties, the National Association of Dental Examiners, and the National Dental Association. These three meet at Niagara Falls, a place that has become historic in dentistry, for it was there the American Dental Association began its labors, and, naturally, the old members of that body still have connected with it many sacred memories.

The Association of Dental Faculties will have before it several questions, the most important of which will probably be that of arranging for the four years' course, beginning 1903. While this, in a measure, must of necessity be left to each individual school, a general consensus of opinion will be required to enable the various colleges to come into an harmonious curriculum. The relations of the dental colleges of this country with those of other lands is a difficult problem, and until finally settled, if this desirable condition should ever be reached, there will be more or less friction, and this, while not always pleasant, is part of an experience that must be lived through in order to reach something better and more enduring.

The National Association of Dental Examiners is a body that seems to be growing in influence and power, as it is expected this year that States heretofore holding aloof will be represented in its deliberations.

There is a hope that the National Dental Association may this year demonstrate more completely than heretofore that it is worthy to be called a scientific body. The sections will be expected to present matter worthy the consideration of such an organization. If, however, the usual routine methods are adopted and the members be served with a rehash of matters long since settled, there will be little gained over previous conventions. The indications are, however, that there will be a decided advance and more original work presented. It is unfortunate that this meeting and that of the American Dental Society of Europe, to be held August 12 to 15, together with the International Dental Federation, meeting at the same time at Stockholm, come so near together. This was unavoidable, as climatic conditions would make a later meeting at Stockholm unsatisfactory. The number from the United States who expect to meet with these two bodies in that city will, it is feared, have a tendency to lessen the interest of the meetings in this country. This has always been the case where there has been a similar conflict of time. The day of adjournment of the National Dental Association, probably Friday, August 1, leaves but a narrow margin of time to reach Stockholm on the 12th.

The meeting of the International Dental Federation is probably the most important of the two gatherings in Sweden, important in the sense of bringing men of many nationalities together. It is questionable whether it will accomplish more than this, for those conversant with conditions existing in various countries have no reason to be sanguine that any real educational harmony will be the outcome of this meeting. While this is true, the final results must mark an advance, if nothing more be accomplished than a better understanding, and this leads naturally to a wider cosmopolitan thought and a broader conception of duty. The meeting, therefore, should be welcomed as a harbinger of a period, yet it may be in the future, but sure to come, when there will be neither English, German, French, Italian, Swedish, or Austrian dentistry, but one profession for the entire civilized world, governed by the same ideas and practical excellencies.

The extension of these meetings over the world in the form of international organizations, both in medicine and in dentistry, will of necessity lead to a better regulation of time of meetings. This journal has for years urged a change of time from the hot months to a period in the spring more conducive to comfort and energy, but as yet there has been no answering voice in favor of a change. If, however, we are to be confronted every year or two with a conflict of time between the home and foreign meetings, to the great injury of the former, there must come a conviction that the present arrangement is neither comfortable nor convenient.

Setting these questions aside, the writer can have only the deepest interest in all such efforts, and will continue to hope that the delegates from America to the two meetings in Stockholm will be able to fully demonstrate that the dentistry of this country is ready and willing to co-operate with that of all other countries upon a basis that will leave each one to work out the problems of dentistry as they arise, in their own way and upon an educational basis best adapted to the needs of the people and the country in which it is practised.

